# Detection of autoantibodies against aquaporin-5 in the sera of patients with primary Sjögren’s syndrome

**DOI:** 10.1007/s12026-016-8786-x

**Published:** 2016-01-19

**Authors:** Jehan Alam, Jung Hee Koh, Nahyun Kim, Seung-Ki Kwok, Sung-Hwan Park, Yeong Wook Song, Kyungpyo Park, Youngnim Choi

**Affiliations:** Department of Oral Microbiology and Immunology, School of Dentistry and Dental Research Institute, Seoul National University, 101 Daehak-ro, Jongno-gu, Seoul, 03080 Republic of Korea; Seoul St. Mary’s Hospital, The Catholic University of Korea, Seoul, Republic of Korea; College of Medicine, Seoul National University, Seoul, Republic of Korea; Department of Oral Physiology, School of Dentistry, Seoul National University, Seoul, Republic of Korea

**Keywords:** Autoimmune disease, Biomarkers, Exocrine dysfunction, Indirect immunofluorescence assay, Sensitivity and specificity, Xerostomia

## Abstract

The pathophysiology of exocrine dysfunction observed in Sjögren’s syndrome (SS) is not fully understood. The purpose of this study was to investigate whether autoantibodies against human AQP5 are present in the sera of SS patients. Frozen sections of mouse submandibular salivary glands, CHO cells over-expressing a human AQP5–GFP fusion protein or GFP, and MDCK cells over-expressing AQP5 were used in the indirect immunofluorescence assay to detect anti-AQP5 autoantibodies in the sera from patients with primary SS. The lysates of HEK-293 cells over-expressing the AQP5–GFP fusion protein or GFP were used for immunoprecipitation. Serum IgG from the SS patients but not from the control subjects stained acinar cells in the mouse salivary glands, the signals of which colocalized with those of AQP5-specific antibodies. Serum IgG from the SS patients also selectively stained AQP5–GFP expressed in CHO cells. However, both the control and SS sera immunoprecipitated the AQP5–GFP, suggesting that autoantibodies against AQP5 were also present in the control sera. The screening of 53 control and 112 SS samples by indirect immunofluorescence assay using the AQP5-expressing MDCK cells revealed the presence of significantly higher levels of anti-AQP5 IgG in the SS samples than in the control samples with sensitivity of 0.73 and a specificity of 0.68. Furthermore, the presence of anti-AQP5 autoantibodies was associated with low resting salivary flow in SS patients. In conclusion, anti-AQP5 autoantibodies were detected in the sera from SS patients, which could be a novel biomarker of SS and provide new insight into the pathogenesis of SS.

**Electronic supplementary material:**

The online version of this article (doi:10.1007/s12026-016-8786-x) contains supplementary material, which is available to authorized users.

## Introduction

Sjögren’s syndrome (SS) is an autoimmune disorder that primarily targets the salivary and lacrimal glands, leading to dryness of the mouth and eyes [1]. In addition to glandular dysfunction, SS patients often present with extra-glandular manifestation, such as tubulointerstitial nephritis, primary biliary cirrhosis, autoimmune cholangitis, autoimmune hepatitis, interstitial lung disease, and the development of lymphocytic malignancies [2–7]. The disease may occur as the primary condition or as a secondary phenomenon in association with other autoimmune diseases, including rheumatoid arthritis, systemic lupus erythematosus, and progressive systemic scleroderma [8]. SS is a multifactorial disease: environmental factors trigger immune responses that damage the salivary and lacrimal gland cells in individuals with a genetic predisposition to the disease [1].

Glandular dysfunction had been understood as a result of apoptotic destruction of acinar cells by infiltrated cytotoxic T cells because the salivary and lacrimal glands of SS patients present abundant lymphocytic infiltration and atrophy [1]. However, the results of more recent studies suggest that glandular dysfunction precedes glandular atrophy. For example, many SS patients with no glandular function retain substantial amounts of intact acinar tissues in their salivary glands. Furthermore, those acinar tissues regain functionality in vitro [9]. The paradigm for glandular hypofunction in SS has thus been shifted toward interference with the secretion process. The control of salivary and lacrimal secretion is governed by muscarinic nerves through the type 3 muscarinic acetylcholine receptor (M3R). Although anti-M3R autoantibody-mediated inhibition of M3R function partially explains the dysfunction of the acinar cells [10], the pathophysiology of exocrine dysfunction observed in SS is not fully understood.

Aquaporin-5 (AQP5), a major water channel protein expressed in the lacrimal and salivary glands, has a critical role in tear and saliva secretion [11]. AQP5-deficient mice secrete hypertonic saliva with a substantially reduced volume [12], which is attributed to a decrease in the water permeability of salivary acini [13]. Anti-AQP4 autoantibodies are detected in patients with neuromyelitis optica, and the presence of anti-AQP4 IgG in the sera is used as a gold standard for the differential diagnosis of neuromyelitis optica from multiple sclerosis [14]. We hypothesized that SS patients may have autoantibodies against AQP5. The purpose of this study was to investigate whether autoantibodies against human AQP5 are present in the sera of SS patients.

## Materials and methods

### Serum samples

This study was done in compliance with the Helsinki Declaration after approvals from the Institutional Review Board of Seoul National University Hospital (IRB Number: 0912-011-302), the Institutional Review Board of Seoul National University School of Dentistry (IRB Number: S-D20140022), and the Institutional Review Board of Seoul St. Mary’s Hospital (IRB Number: KC13ONMI0646). The study also conformed to the STROBE guidelines. For this pilot case–control study, serum samples were obtained from two groups of patients: (1) 10 primary SS patients enrolled at the Rheumatology Clinic, Seoul National University Hospital, who were diagnosed according to the 2002 American-European Consensus group (AECG) classification criteria for primary Sjögren’s syndrome [15] and (2) 102 primary SS patients enrolled at the Korean Initiative of primary Sjögren’s Syndrome (KISS) who fulfilled the 2002 American-European Consensus group (AECG) classification criteria and/or the 2012 American College of Rheumatology (ACR) criteria [16]. The samples included in the pooled sera were chosen based on the availability. All samples were obtained before starting treatment. In addition, resting and stimulated whole salivary flow rates were measured by spiting and masticatory stimuli using wax gum (GC America Inc, St. Alsip, IL, USA), respectively, according to the method previously described [17]. All patients were females with ages ranging from 21 to 80 years (mean age 52.5 ± 10.7 years). Control sera were obtained from 53 healthy female controls who did not show any signs of SS symptoms (mean age 37.1 ± 7.4 years). Subjects with systemic disease other than hypertension were excluded. Written informed consent was obtained from all the subjects.

### Cell culture

All cell lines were obtained from Korean Cell Line Bank (Seoul, Korea). Chinese hamster ovary (CHO) cells were cultured in F-12 medium containing 10 % FBS and 1 % penicillin/streptomycin, while human embryonic kidney (HEK)-293 cells and Madin-Darby canine kidney (MDCK) cells were maintained in DMEM medium with 10 % FBS and 1 % penicillin/streptomycin.

### AQP5-encoding constructs and transfection of cells

The AQP5 cDNA was cloned into a pEGFP-N1 vector (Clontech, Mountain View, CA, USA) using XhoI and BamHI sites and into a pcDNA3.1 vector (Invitrogen, Carlsbad, CA, USA) using BamHI and XhoI restriction sites. The cloned plasmids were transfected into cells with the calcium phosphate precipitation method [18].

### Indirect immunofluorescence assay (IIFA)

For IIFA, all tissues and cells were fixed with 4 % paraformaldehyde in PBS pH 7.4, subjected to antigen retrieval by incubation in sodium citrate buffer pH 6 at 105 °C for 20 min, and then permeabilized with 0.3 % Triton X-100 before blocking and incubation with primary antibodies, which gave the best result by staining with the commercial anti-AQP5 antibodies.

The use of animal tissues followed a protocol approved by the Seoul National University Animal Care and Use Committee. After stimulating 12-week-old C57BL/6 mice (Orient Bio Inc., Seongnam, Gyeonggi, Korea) with 5 μg/g body weight of pilocarpine (Sigma-Aldrich Korea, Seoul, Korea), the submandibular glands were removed and fixed with 4 % paraformaldehyde in PBS pH 7.4 overnight. The fixed tissues were immersed in 30 % sucrose–PBS pH 7.4 and embedded in the OCT compound. Cryostat sections were mounted on saline-coated glass slides (Muto pure chemicals, Tokyo, Japan) and air-dried. After antigen retrieval and permeabilization, the sections were blocked with 5 % BSA in PBS and were then incubated with goat anti-AQP5 antibodies (Santa Cruz, Paso Robles, CA, USA) and either the pooled SS or pooled control sera (1:200 dilution) overnight, followed by Alexa Fluor 488-conjugated donkey anti-goat IgG (Invitrogen) and CF™ 594-conjugated rabbit anti-human IgG antibodies (Sigma-Aldrich).

CHO cells cultured on collagen-coated glass slides were transfected with either pEGFP-N1-AQP5 or pEGFP-N1. MDCK cells were transfected with pcDNA3.1-AQP5. The transfected CHO cells were incubated with rabbit anti-GFP antibodies (Sant Cruz) and either the pooled control or the pooled SS sera (1:200 dilution), followed by Alexa Fluor 488-conjugated goat anti-rabbit and Alexa Fluor 555-conjugated goat anti-human IgG (Invitrogen). The transfected MDCK cells were incubated with goat anti-AQP5 and various dilutions of either the control or SS sera, followed by Alexa Fluor 488-conjugated donkey anti-goat IgG and CF™ 594-conjugated rabbit anti-human IgG. To detect human IgA, Alexa flour 594-conjugated rabbit anti-human IgA antibodies (Jackson ImmunoResearch, West Grove, PA, USA) were used. All images were taken with a confocal microscope LSM 700 (Carl Zeiss, Jena, Germany). In the case of the stained MDCK–AQP5 cells, three areas of AQP5-expressing cells were randomly selected and sequentially imaged for the presence of anti-AQP5 IgG/IgA. Because many SS samples had stained nuclei as well as AQP5, the relative intensities of the anti-AQP5 signals were determined by decreasing the brightness of the red signal until the signals of the anti-AQP5 IgG/IgA disappeared. The mean of the ∆ brightness obtained in three images was used to express the level of anti-AQP5 IgG/IgA for each sample.

### Immunoprecipitation and western blot

HEK-293 cells were transfected with pAQP5-EGFP or pEGFP-N1. Forty-eight hours after transfection, cells were lysed with lysis buffer (40 mM octyl-β-d-1-thioglucopyranoside, 50 mM Tris–HCl, and 150 mM NaCl, pH 7.4). Proteins (250 µg of cell lysates) were incubated with 1 μg anti-AQP5 antibodies, pooled control sera, or pooled SS sera overnight, followed by precipitation with protein A agarose beads (Pierce Biotechnology, Rockford, IL, USA). After washing, the beads were resuspended in Laemmli sample buffer containing β-mercaptoethanol and incubated at 70 °C for 15 min. The proteins were separated on a 12 % SDS-PAGE gel, transferred to PVDF membrane (Millipore, Billerica, MA, USA), and immunoblotted with goat anti-AQP5 or rabbit anti-GFP antibodies.

### Statistics

With the levels of anti-AQP5 IgG/IgA detected in the SS and control groups, a receiver operating characteristic (ROC) analysis by a nonparametric method and the Mann–Whitney *U* test were performed. Associations between measures of salivary rate and the presence of autoantibodies were examined using one-way analysis of variance. Because some groups did not pass the normality test, the difference was also analyzed by Mann–Whitney *U* test. All statistics were performed using SPSS (SPSS Inc., Chicago, USA).

## Results

### Serum IgG from SS patients stain AQP5 in mouse salivary glands

**Fig. 1:**
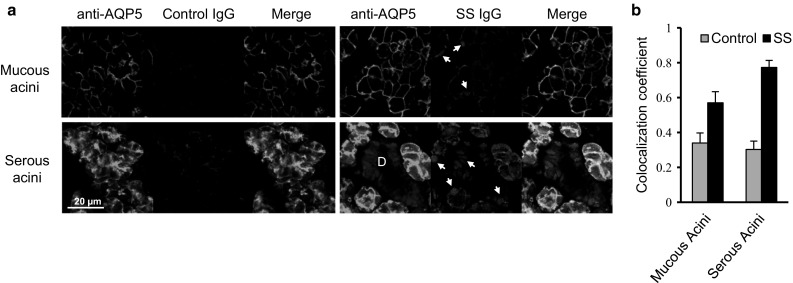
Serum IgG from SS patients co-localizes with AQP5 in the mouse salivary glands. **a** Sections of the mouse submandibular gland were stained with goat anti-AQP5 antibodies and either the pooled control or pooled SS sera followed by Alexa Fluor 488-conjugated anti-goat IgG and CF™ 594-conjugated anti-human IgG. The areas of mucous and serous acini were imaged with confocal microscopy. *D* duct; *arrows* nuclei. **b** The colocalization of *green* and *red* signals in five images were calculated with Mander’s coefficient (Color figure online)

To investigate the presence of autoantibodies against AQP5 in the sera of SS patients, sections of mouse salivary glands were dual-stained with the pooled sera of patients (*n* = 4) and AQP5-specific goat IgG that targets the cytoplasmic tail of murine, rat, and human AQP5. The amino acid sequence of AQP5 is highly conserved between humans and mice with 91 % identities and 96 % homology. AQP5 expression was observed at the apical site of the serous acinar cells in the highest levels and also at the basolateral site of both the mucous and serous acinar cells but not in the ductal cells, as previously reported [19]. While the pooled sera of control subjects (*n* = 4) barely stained the mouse salivary glands, the SS sera strongly stained the nuclei and plasma membranes of the acinar cells (Fig. 1a). The signals of SS IgG showed a high degree of colocalization with those of AQP5-specific goat IgG, which was confirmed with Mander’s overlap coefficient (Fig. 1b).

### Serum IgG from SS patients selectively stain AQP5–GFP-transfected cells

**Fig. 2:**
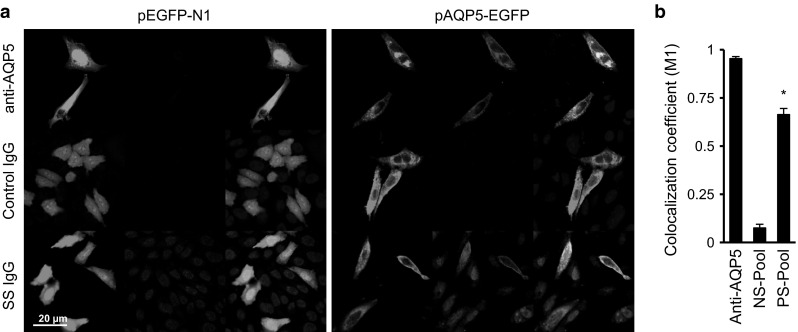
Serum IgG from SS patients selectively stains AQP5–GFP-transfected cells. **a** CHO cells were transfected with either pEGFP-N1 or pAQP5-EGFP. The transfected cells were stained with goat anti-AQP5 antibodies, control sera, or SS sera (*red*) together with anti-GFP antibodies (*green*). **b** The colocalization of *green* and *red* signals in 18 images were calculated with Mander’s coefficient (Color figure online)

Because SS patients may have autoantibodies against other proteins expressed in the salivary gland, the specificity of the autoantibodies was further investigated with CHO cells transfected with a transgene encoding a human AQP5–GFP fusion protein or GFP alone. While the GFP was dispersed throughout the cytoplasm and nuclei, AQP5–GFP was localized mostly to the plasma membranes and vesicular organelles. The anti-AQP5 goat IgG stained the CHO cells expressing AQP5–GFP but not those expressing GFP alone. The pooled SS sera stained the nuclei of the CHO cells regardless of the type of transgene but also specifically stained the cells expressing AQP5–GFP. The pooled control sera did not stain either GFP- or AQP5–GFP-expressing cells (Fig. 2a). The signals of both anti-AQP5 goat IgG and SS IgG overlapped with the signal of GFP localized to the plasma membrane and intracellular vesicles in the AQP5–GFP-expressing cells, which was confirmed with Mander’s overlap coefficient (Fig. 2b).

### Both the control and SS sera immunoprecipitate AQP5–GFP

**Fig. 3:**
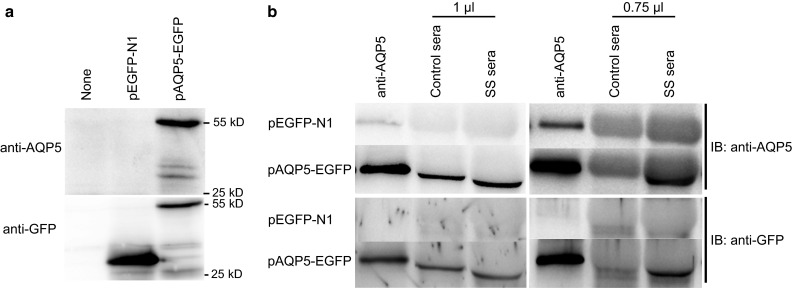
Both the control and SS sera immunoprecipitate AQP5–GFP. HEK-293 cells were transfected with either pEGFP-N1 or pAQP5-EGFP. **a** Lysates of non-transfected and transfected HEK-293 cells were separated by SDS-PAGE and immunoblotted with anti-AQP5 or anti-GFP antibodies. **b** Lysates of HEK-293 cells transfected with either pEGFP-N1 or pAQP5-EGFP were incubated with anti-AQP5 antibodies, control sera, or SS sera. The immune complexes precipitated with protein A agarose beads were immunoblotted with either anti-AQP5 or anti-GFP antibodies

The selective staining of AQP5–GFP but not of GFP by the SS sera suggests that AQP5 acts as an autoantigen in the SS patients. However, colocalization, i.e., co-occurrence of two fluorophores in the same pixel, does not necessarily mean that the two signals target the same molecule. Therefore, the specific binding of SS IgG to AQP5 was further studied by immunoprecipitation. HEK-293 cells were transfected with either pEGFP-N1 or pAQP5-GFP which express the 27 KD GFP and 55 KD AQP5–GFP proteins, respectively (Fig. 3a). The anti-AQP5 IgG precipitated the AQP5–GFP, which was not precipitated from the lysates of the HEK-293 cells expressing GFP alone. Both the control and SS sera precipitated the AQP5–GFP, suggesting the presence of autoantibodies against AQP5 not only in the SS sera but also in the control sera (Fig. 3b left panel). When decreased amounts of sera were used, however, only the SS sera precipitated AQP5–GFP (Fig. 3b right panel). In an additional blinded experiment using several control and SS sera, all samples immunoprecipitated AQP5–GFP (data not shown).

### Higher levels of anti-AQP5 IgG and IgA were detected in the SS sera by IIFA

**Fig. 4:**
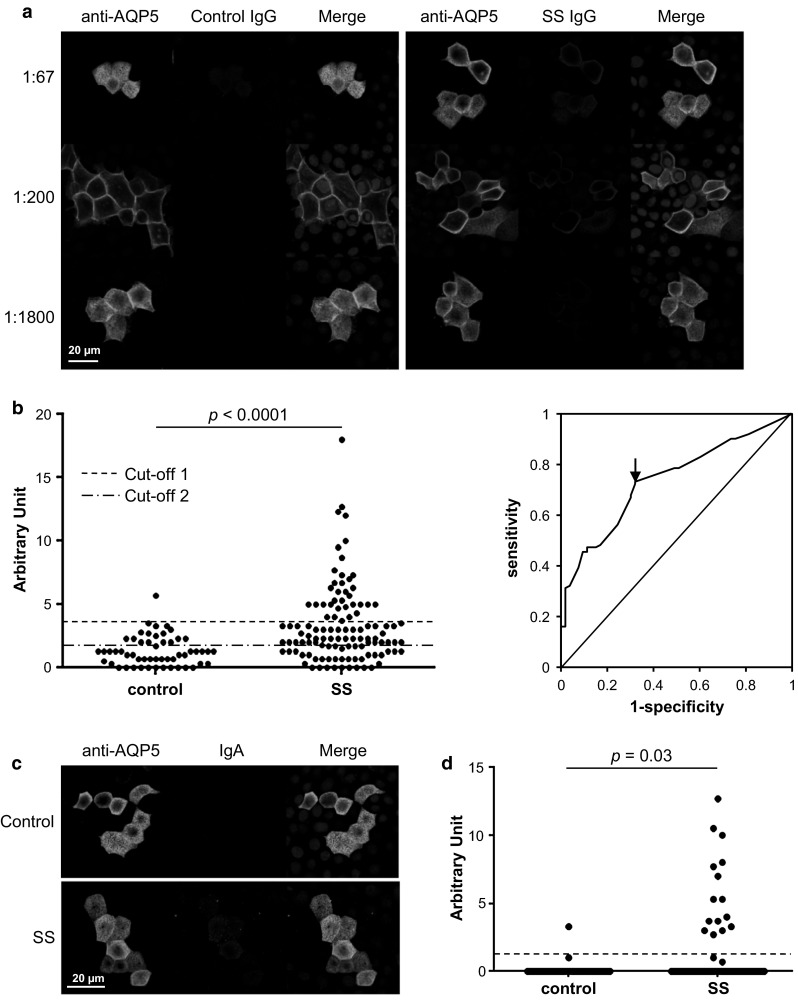
Higher levels of anti-AQP5 IgG and IgA were detected in the SS sera by IIFA. **a** MDCK cells over-expressing AQP5 were stained with anti-AQP5 antibodies and various dilutions of either the control or SS sera, followed by Alexa Fluor 488-conjugated anti-goat IgG (*green*) and CF™ 594-conjugated anti-human IgG (*red*). **b** The intensities of the *red* signals for anti-AQP5 IgG were expressed by the magnitude of brightness that was reduced until the staining of AQP5 disappeared. A ROC curve for the levels of anti-AQP5 IgG is shown. The *arrow* indicates the value used for cutoff 2 in the *left panel*. **c** MDCK cells over-expressing AQP5 were stained with anti-AQP5 antibodies and either the control or SS sera (1:20 dilution), followed by Alexa Fluor 488-conjugated anti-goat IgG (*green*) and Alexa Fluor 555-conjugated anti-human IgA (*red*). **d** The intensities of the *red* signals for anti-AQP5 IgA were expressed by the magnitude of brightness that was reduced until the staining of AQP5 disappeared (Color figure online)

Currently, the standard method to detect anti-AQP4 autoantibodies for the diagnosis of neuromyelitis optica is with IIFA [20]. Therefore, a similar cell-based IIFA was developed by over-expressing AQP5 in MDCK cells which have been widely used in studies on AQP5 function [21, 22]. The staining protocol was first optimized with the pooled sera. Both the SS and control sera contained anti-AQP5 IgG; however, a clear difference in their titers was observed (Fig. 4a). The 1:200 dilution was chosen to screen 53 control and 112 SS samples for anti-AQP5 IgG. The anti-AQP5 IgG was detected in 43 control and 103 SS samples with a significant difference in their intensities (*p* < 0.0001). When the cutoff value was set as the mean + 2 SD of the control values, 35 (31.3 %) SS samples were positive for the anti-AQP5 IgG (Fig. 4b left panel, cutoff 1). When the cutoff value at which the accuracy was the highest was chosen from the ROC curve (Fig. 4b right panel), 17 (32.1 %) control and 82 (73.2 %) SS samples were positive for the anti-AQP5 IgG, resulting in a sensitivity of 0.73 and a specificity of 0.68 (Fig. 4b left panel, cutoff 2). We also screened the samples for the presence of anti-AQP5 IgA with a 1:20 dilution (Fig. 4c). The anti-AQP5 IgA was detected in only one (1.9 %) control and 15 (13.4 %) SS samples, where the cutoff was set as the mean + 2 SD of the control values (Fig. 4d). All the anti-AQP5 IgA-positive samples also contained anti-AQP5 IgG, although the levels of anti-AQP5 IgG did not reach the cutoff 2 in four SS samples. Therefore, total 86 (76.8 %) SS samples were positive for either anti-AQP5 IgG or IgA.

### The presence of anti-AQP5 autoantibodies was associated with low salivary flow rate

Associations between the presence of anti-AQP5 IgG (by cutoff 2) or IgA autoantibodies and salivary flow rates were examined in patients for whom the salivary flow rates were recorded at the time of sampling. The presence of anti-AQP5 IgG was associated with low resting salivary flow but not with stimulated salivary flow. The patients with anti-AQP5 IgA presented lower levels of both resting and stimulated salivary flow than those without the autoantibodies. However, significance was achieved for only resting salivary flow (Table 1 and Supplementary Table 1). Importantly, the presence of either anti-Ro or anti-La antibodies was not associated with the levels of salivary flow (Table 1 and Supplementary Table 1).

**Table 1:** Association between whole salivary flow and the presence of anti-AQP5 autoantibodies in SS patients

Presence of autoantibodies	No. of patients	Mean salivary flow^a^ (95 % CI)	*P*
Anti-AQP5 IgG		Resting	
−	28	0.097 (0.045, 0.149)	0.003^b^
+	63	0.050 (0.031, 0.069)	0.147^c^
		Stimulated	
−	27	0.554 (0.362, 0.747)	0.697^b^
+	61	0.511 (0.389, 0.633)	0.717^c^
Anti-AQP5 IgA		Resting	
−	79	0.070 (0.047, 0.093)	0.048^b^
+	12	0.027 (-0.011, 0.064)	0.010^c^
		Stimulated	
−	76	0.556 (0.444, 0.667)	0.057^b^
+	12	0.326 (0.110, 0.543)	0.089^c^
Anti-Ro IgG		Resting	
−	9	0.040 (0.002, 0.149)	0.445^b^
+	82	0.067 (0.044, 0.090)	0.512^c^
		Stimulated	
−	8	0.482 (0.238, 0726)	0.793^b^
+	80	0.529 (0.419, 0.638)	0.800^c^
Anti-La IgG		Resting	
−	42	0.066 (0.042, 0.091)	0.851^b^
+	49	0.062 (0.029, 0.096)	0.288^c^
		Stimulated	
–	40	0.550 (0.407, 0.692)	0.648^b^
+	48	0.503 (0.357, 0.649)	0.508^c^

^a^ml/min

^b^ANOVA

^c^Mann–Whitney *U* test

## Discussion

The results of this study showed that autoantibodies against AQP5 are present in the sera of SS patients. Furthermore, patients with anti-AQP5 autoantibodies presented significantly lower resting salivary flow than those without the anti-AQP5 antibodies. This is the first report on the association between anti-AQP5 autoantibodies and SS. Like other AQPs, AQP5 consists of six transmembrane alpha helixes connected by five loops, and the protein forms a water channel as narrow as 1–4 Å through the plasma membrane [23]. If antibodies bind to the extracellular loops of AQP5, the antibodies may block the passage of water molecules through the channel. AQP5 is mainly localized to the apical membrane of acinar cells and intercalated ducts. AQP5 has also been found at the basolateral membrane of acinar cells in the mouse salivary glands [19]; however, the basolateral localization of AQP5 in normal human salivary glands has not been reported yet. Although dyslocalization of AQP5 from the apical to the basolateral sites of acinar cells in SS patients has been reported by two groups, the other two groups reported no difference in the subcellular localization of AQP5 between the normal and SS salivary glands [24–27]. AQP5 located on the basolateral side can be accessed by anti-AQP5 autoantibodies present in the tissue fluid, while AQP5 present on the apical side of acinar cells can be accessed by anti-AQP5 autoantibodies present in saliva.

Unexpectedly, the anti-AQP5 autoantibodies were also detected in the sera of many healthy controls at low levels. Highly conserved AQPs are distributed throughout all the kingdoms of life, including bacteria. It has been shown that mouse immune serum raised against *Escherichia coli* AQPZ react with human AQP4 [28]. When the bacterial protein database was BLAST-searched using the human AQP5 sequence as a query, AQPZ or porins from many human-associated bacteria had a high degree of homology with AQP5 (Supplementary Table 2). The human-associated bacteria containing the AQP5-homologous proteins included infectious pathogens (*Yersinia enterocolitica*, *Vibrio vulnificus*, *Vibrio parahaemolyticus*, etc.), opportunistic pathogens (*Acinetobacter baumannii*, *Enterococcus faecalis*, *E. coli*, *Streptococcus pneumoniae*, etc.), and the members of normal flora (*Enterobacter cloacae*, *Neisseria subflava*, *Streptococcus oralis*, etc.). Therefore, there is a possibility that the autoantibodies against AQP5 were developed during an immune response against bacterial proteins. While IIFA showed a clear difference in the levels of anti-AQP5 autoantibodies between the control and SS sera, immunoprecipitation was not effective in differentiating the control and patient samples. This discrepancy could be attributed to potential differences in the conformations and exposed epitopes of AQP5 present in membrane versus lysates. Sequence alignment of the selected bacterial AQPs with human AQP5 revealed that sequences are conserved mostly at the transmembrane alpha helixes and the water channel-forming loops *B* and *E* (Supplementary Figure). In this regard, the anti-AQP5 autoantibodies detected in the control and SS samples could have different effects such as inhibiting the function of AQP5. Unfortunately, the salivary flow rates were available only for 10 control subjects, and the effect of the anti-AQP5 autoantibodies on salivary flow in the control group could not be evaluated. A cell-based functional assay to evaluate the effect of anti-AQP5 autoantibodies on water passage through AQP5 is currently under development, which will provide direct evidence for the role of anti-AQP5 autoantibodies in the salivary flow. Another limitation of the current study is a relatively small sample size. Therefore, further studies using samples from larger SS patient cohorts and diverse control subjects, including other autoimmune diseases, are needed.

The presence of anti-Ro/SSA and/or anti-La/SSB autoantibodies in serum is a diagnostic hallmark of SS [15]. A number of other autoantibodies such as anti-salivary gland protein 1, anti-carbonic anhydrase 6, anti-parotid secretory protein, anti-α-fodrin, anti-M3R, anti-nuclear, and anti-smooth muscle antibodies have been identified in SS [5, 6, 29]. Except for the anti-parotid secretory protein and anti-M3R antibodies, most autoantibodies target antigens that are normally present inside cells. Therefore, those autoantibodies reflect the apoptotic destruction of gland tissues, that is, a result of the disease process rather than the cause of the disease [10]. The degree of salivary dysfunction in SS patients does not correlate with the degree of glandular tissue destruction [30]. Indeed, the presence of either anti-Ro or anti-La autoantibodies was not associated with salivary hypofunction in the current study.

In contrast, anti-M3R autoantibodies have the potential to interfere with the secretory process by inhibiting signaling through M3R and AQP5 translocation [29, 31, 32]. The binding of autoantibodies to M3R also down-regulates the receptors from the plasma membrane by inducing internalization [33]. However, the functional data have not been reconciled with the sensitivity and specificity of the anti-M3R autoantibodies in screening trials [10]. Although anti-M3R autoantibodies have been detected in 9–100 % of SS patients depending on the method and antigens used [34], a recent meta-analysis study concluded that the anti-M3R antibody has high specificity (0.95) but relatively low sensitivity (0.43) to diagnose SS [35]. In this aspect, the anti-AQP5 autoantibodies identified in the current study could complement anti-M3R autoantibodies.

In conclusion, anti-AQP5 autoantibodies were detected in the sera of SS patients, which could be a novel biomarker of SS and provide new insight into the pathogenesis of SS. In addition, association between the presence of anti-AQP5 autoantibodies and resting salivary flow in SS patients suggests its potential as a biomarker that reflects disease activity.

## Electronic supplementary material

Below is the link to the electronic supplementary material.

Supplementary material 1 (PPTX 119 kb)

Supplementary material 2 (XLSX 15 kb)

Supplementary material 3 (XLSX 275 kb)
