# Supplementary material for: Detection of autoantibodies against aquaporin-5 in the sera of patients with primary Sjögren’s syndrome
Source: Immunol Res. 2016 Jan 19;64:848–56. doi: 10.1007/s12026-016-8786-x (PMC4930796; doi:10.1007/s12026-016-8786-x)
Supplement: Supplementary file 1 — Supplementary material 1 (PPTX 119 kb) [file 12026_2016_8786_MOESM1_ESM.pptx]

## Slide 1
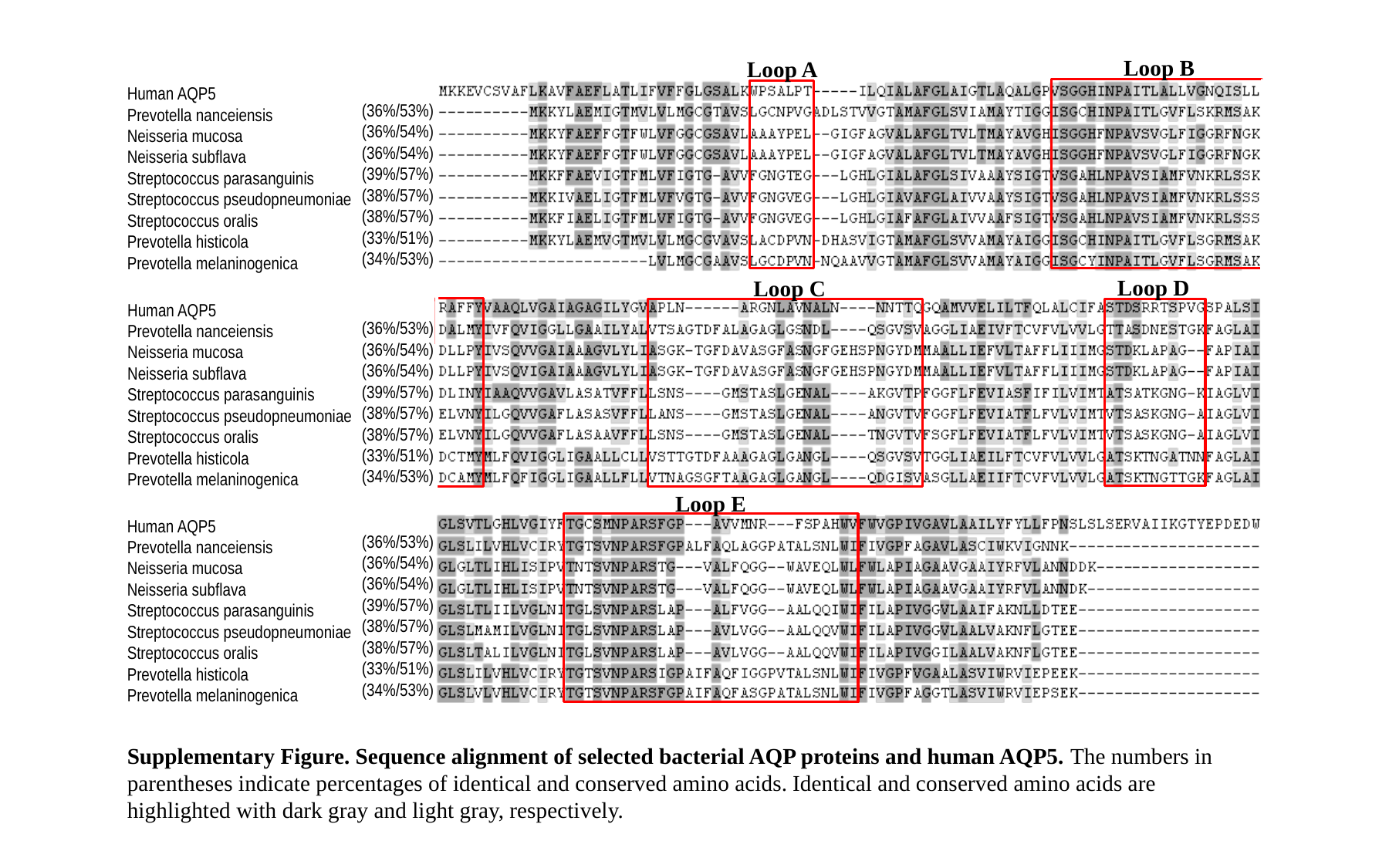

Loop B
Loop A
Loop D
Loop C
Loop E
Human AQP5
Prevotella nanceiensis
Neisseria mucosa
Neisseria subflava
Streptococcus parasanguinis
Streptococcus pseudopneumoniae
Streptococcus oralis
Prevotella histicola
Prevotella melaninogenica
(36%/53%)
(36%/54%)
(36%/54%)
(39%/57%)
(38%/57%)
(38%/57%)
(33%/51%)
(34%/53%)
Human AQP5
Prevotella nanceiensis
Neisseria mucosa
Neisseria subflava
Streptococcus parasanguinis
Streptococcus pseudopneumoniae
Streptococcus oralis
Prevotella histicola
Prevotella melaninogenica
(36%/53%)
(36%/54%)
(36%/54%)
(39%/57%)
(38%/57%)
(38%/57%)
(33%/51%)
(34%/53%)
Human AQP5
Prevotella nanceiensis
Neisseria mucosa
Neisseria subflava
Streptococcus parasanguinis
Streptococcus pseudopneumoniae
Streptococcus oralis
Prevotella histicola
Prevotella melaninogenica
(36%/53%)
(36%/54%)
(36%/54%)
(39%/57%)
(38%/57%)
(38%/57%)
(33%/51%)
(34%/53%)
Supplementary Figure. Sequence alignment of selected bacterial AQP proteins and human AQP5. The numbers in parentheses indicate percentages of identical and conserved amino acids. Identical and conserved amino acids are highlighted with dark gray and light gray, respectively.
